# Effects of sleep quality on the association between problematic internet use and quality of life in people with substance use disorder

**DOI:** 10.1192/bjo.2022.557

**Published:** 2022-08-10

**Authors:** Mohsen Saffari, Hsin-Pao Chen, Ching-Wen Chang, Chia-Wei Fan, Shih-Wei Huang, Jung-Sheng Chen, Kun-Chia Chang, Chung-Ying Lin

**Affiliations:** Health Research Center, Lifestyle Institute, Baqiyatallah University of Medical Sciences, Tehran, Iran; and Health Education Department, School of Health, Baqiyatallah University of Medical Sciences, Tehran, Iran; Division of Colon and Rectal Surgery, Department of Surgery, E-Da Hospital, Kaohsiung, Taiwan; and School of Medicine, College of Medicine, I-Shou University, Kaohsiung, Taiwan; Graduate Institute of Social Work, National Taiwan Normal University, Taipei, Taiwan; Department of Occupational Therapy, AdventHealth University, Florida, USA; Institute of Environmental Toxin and Emerging Contaminant, Cheng Shiu University, Kaohsiung, Taiwan; and Center for Environmental Toxin and Emerging-Contaminant Research, Cheng Shiu University, Kaohsiung, Taiwan; Department of Medical Research, E-Da Hospital, Kaohsiung, Taiwan; Department of General Psychiatry, Jianan Psychiatric Center, Ministry of Health and Welfare, Tainan, Taiwan; and Department of Natural Biotechnology, Nan Hua University, Chiayi, Taiwan; Institute of Allied Health Sciences, College of Medicine, National Cheng Kung University, Tainan, Taiwan; Biostatistics Consulting Center, National Cheng Kung University Hospital, College of Medicine, National Cheng Kung University, Tainan, Taiwan; Department of Public Health, College of Medicine, National Cheng Kung University, Tainan, Taiwan; and Department of Occupational Therapy, College of Medicine, National Cheng Kung University, Tainan, Taiwan

**Keywords:** Internet addiction, substance misuse, mediating effect, quality of life, sleep

## Abstract

**Background:**

Problematic internet use, especially in people with substance use disorder, may negatively affect their quality of life (QoL). However, it is unclear whether sleep quality is a key mediator in the association between problematic internet use and QoL among people with substance use disorder.

**Aims:**

This study aimed to investigate the relationship between problematic internet use and QoL and how sleep quality may mediate the association between these two variables.

**Method:**

Overall, 319 people (85% male) with substance use disorder (mean age 42.2 years, s.d. 8.9) participated in a cross-sectional study in Taiwan. The Smartphone Application-Based Addiction Scale, Bergan Social Media Addiction Scale, Internet Gaming Disorder-Short Form, Pittsburgh Sleep Quality Index and World Health Organization Quality of Life Questionnaire Brief Version were used.

**Results:**

The prevalence of sleep problems was 56%. There were significant and direct associations between sleep quality and two types of problematic internet use, and between sleep quality and different dimensions of QoL. All types of problematic internet use were significantly and negatively correlated with QoL. Mediated effects of sleep quality in relationships between the different types of problematic internet use and all dimensions of QoL were significant, except for problematic use of social media.

**Conclusions:**

Different types of problematic internet use in people with substance use disorder may be directly associated with reduced QoL. Sleep quality as a significant mediator in this association may be an underlying mechanism to explain pathways between problematic internet use and QoL in this population.

According to the DSM-5, substance use disorder (SUD; formerly called substance/drug addiction) could involve the use of a wide range of substances, including alcohol, stimulants, cannabis and opioids, which cause an individual to continue using despite its negative effects on physical and mental health. The DSM-5 suggests several diagnostic criteria for SUD, such as tolerance, withdrawal syndrome, risky use, having related individual/social problems, decreased attention for fulfilling social roles, prolonged and increased frequency of usage, multiple recurrences, related bio-psychological problems and the urge to use the substance.^1^ SUD is a persistent condition and may lead to numerous treatment and relapse cycles for affected people.^2^ These people, especially those who use injectable drugs, are at higher risk for engagement with subsequent mental or physical problems. For example, depression and mood disorders are prevalent in this population; and they are more susceptible to infectious diseases (e.g. various types of viral hepatitis and infection with HIV) than the general population, because of their risky behaviours.^3,4^ The World Drug Report 2021 estimated that more than 270 million people worldwide had used drugs illegally in the past year; around 36 million people had SUD.^5^ The National Center for Drug Abuse Statistics suggests that nearly 12% of Americans aged 12 years and older may be classified as illegal drug users. If including alcohol and tobacco misuse, more than 60% of people aged over 12 years may be considered misusers of addictive substances.^6^ The statistics indicate that SUD is prevalent in developed countries and developing regions, including South-East Asia.^7^ For example, a Taiwanese study found a prevalence rate of 11% for SUD in adolescents.^8^

## Problematic internet use in people with SUD

Problematic internet use (PIU), also known as ‘internet addiction’ or ‘pathological internet use’, is defined as a continuous and recurrent internet use that causes addictive symptoms, such as irritability when not being online, inattention to time when using the internet (tolerance), preoccupation and withdrawal-related mood fluctuations.^9,10^ The PIU involves various types of excessive internet use, such as frequently downloading, spending considerable time for social networking or internet shopping, immoderate smartphone use for online purposes and prolonged online gaming.^11^ Studies have shown that PIU may influence between 10 and 30% of different regions across the world.^12–14^ In particular, youth populations with PIU may experience social isolation, poor social support and lower social skills.^13^ Several PIU-related risk factors have been identified, including male gender, early accessibility, poverty, living in rural regions, impulsivity and low self-esteem personality traits.^15,16^

According to problem behaviour theory, the problematic/addictive behaviours tend to be intercorrelated and may happen concurrently.^17^ For example, behaviours such as social media addiction, pathological gambling, food addiction, mobile phone addiction and PIU have been identified as significant correlators of substance misuse.^18–21^ These addictive behaviours usually are associated with self-control inability, which interferes with other activities of daily living and physiological events, such as the circadian rhythm and sleep quality.^22^ Studies have indicated that people with SUDs are at higher risk for PIU than the general population; people affected by PIU are more likely to become substance misusers in the future.^19,23^

## Sleep quality in people with SUD

The associations between sleep problems and substance misuse have been established in previous studies.^24^ The research found that higher substance use may cause sleep problems, and any sleep disturbances may propel the individual toward substance use. The co-occurrence of sleep problems and substance misuse, especially in youth and younger adults, is common and may cause severe health issues (e.g. immune system deficiencies, increased car accidents), mental health disorders (e.g. anxiety, depression) and suicidal ideation.^24,25^ Both sleep problems and SUDs can negatively affect physical and psychological well-being, and reduced quality of life (QoL) is recognised as a negative outcome associated with such disorders.^26^ People who experience co-occurrence of these events are at higher risk of chronic diseases such as diabetes, obesity and cardiovascular conditions.^25^ They also have problems in their social communication and interpersonal relationships.^27^

## PIU, sleep quality, QoL and study hypothesis

Compared with the general population, people with SUDs have a lower QoL.^28,29^ Because QoL has been recognised as the key outcome measure to assess the impact of numerous psychological problems (including addictive behaviours) on health and well-being, understanding whether and how PIU is associated with QoL in people with SUDs is important in developing interventions.^26^ A growing number of studies indicate that PIU reduces QoL.^30,31^ However, given that PIU and SUD are associated and that PIU and SUD could negatively affect QoL, the relationship between PIU and QoL in people with SUDs remains unclear. Moreover, previous studies found that PIU is a predictor of sleep disturbances.^32^ The existing literature also documents that sleep problems lead to poor QoL.^26,27^ Based on findings of other populations, it is speculated that PIU is associated with SUD via the mediating effect of sleep quality, although this pathway has not been investigated in individuals with SUDs. Thus, it is unknown whether sleep problems could mediate the relationship between PIU and QoL in those with SUDs. The current study sought to fill these knowledge gaps by investigating the relationship between PIU, sleep quality and QoL in adults with SUDs. We hypothesised that PIU is associated with QoL in people with SUD, and this association is mediated by sleep quality.

## Method

### Participants and procedures

This study employed a cross-sectional design. The authors assert that all procedures contributing to this work comply with the ethical standards of the relevant national and institutional committees on human experimentation and with the Helsinki Declaration of 1975, as revised in 2008. Furthermore, all procedures involving human patients were approved by the Jianan Psychiatric Center (approval number 19-034) Institutional Review Board. All participants signed written informed consent.

Individuals with opioid (involving heroin), amphetamine or alcohol use disorders according to diagnostic criteria in the DSM-5 were recruited from addiction out-patient clinics of Jianan Psychiatric Center in Tainan, Taiwan. Other than the diagnosis of SUDs, the inclusion criteria also included being over 20 years old and having sufficient mental capacity to understand assessment scales used in the study, as assessed by an experienced psychiatrist. In addition, research assistants with health psychology expertise verified sufficient cognition through conversation. Exclusion criteria included having an intellectual disability and having dementia, schizophrenia or other acute substance-induced psychotic disorders.

### Recruitment procedures

First, one psychiatrist screened the potential participants and referred them to the research team. Second, the research assistants introduced the study to potential participants. Those who agreed to participate and gave written informed consent were enrolled. Next, the participants were asked to complete the study questionnaire in a quiet, isolated room, undisturbed, supervised by the research assistants. The average time for completing the questionnaire was about 20 min. After that, the research team engaged in a standardised face-to-face procedure as described by Lavrakas,^33^ to assure the completeness of the questionnaires: (a) the participant handed the completed questionnaire to the research assistant; (b) the research assistant screened all items in the questionnaire to ensure there were no missing answers; (c) if answers were found to be missing, the research assistant alerted the participant to the missing answers and asked the participant to provide them; (d) if the participant refused to complete the missing answers, the research assistant asked the participant to explain their refusal; and (e) if the participant refused because they could not understand the questions, the research assistant clarified the questions enabling the participant to complete the questionnaire. The team member also explained and discussed the questionnaire results with the participants.

### Measures

#### Smartphone Application-Based Addiction Scale

The Smartphone Application-Based Addiction Scale (SABAS) has six items rated on a six-point Likert scale (1 indicating strongly disagree; 6 indicating strongly agree). It examines an individual's level of problematic smartphone usage. A higher SABAS score indicates a higher level of problematic smartphone use, with a cut-off of >21 indicating the presence of problematic smartphone use.^34^ A sample item of the SABAS is ‘My smartphone is the most important thing in my life’. The SABAS has been found to have satisfactory psychometric properties in different language versions (i.e. Chinese, English, Hungarian and Persian).^35–41^ A Chinese version of the SABAS was used in the current study. The internal consistency of the SABAS in the present study was good (α = 0.80).

#### Bergan Social Media Addiction Scale

The Bergan Social Media Addiction Scale (BSMAS) has six items rated on a five-point Likert scale (1 indicating very rarely; 5 indicating very often). It examines an individual's level of problematic social media use. A higher BSMAS score indicates a higher level of problematic social media use, with a cut-off >19 indicating the presence of problematic social media use.^42^ A sample item of the BSMAS is ‘How often during the last week have you spent thinking about social media or planned use of social media?’. The BSMAS has been found to have satisfactory psychometric properties in different language versions (i.e. Chinese, English, Hungarian, Italian and Persian).^35,38,41–48^ A Chinese version of the BSMAS was used in the current study. The internal consistency of the BSMAS in the present study was good (α = 0.87).

#### Internet Gaming Disorder Scale – Short Form

The Internet Gaming Disorder Scale – Short Form (IGDS9-SF) has nine items rated on a five-point Likert scale (1 indicating never; 5 indicating very often). It examines an individual's level of problematic gaming. A higher IGDS9-SF score indicates a higher level of problematic gaming, with a cut-off >32 indicating the presence of problematic gaming.^49^ A sample item of the IGDS9-SF is ‘Do you feel more irritability, anxiety, or even sadness when you try to either reduce or stop your gaming activity?’. The IGDS9-SF has been found to have satisfactory psychometric properties in different language versions (i.e. Chinese, English, Hungarian and Persian).^35,38,50^ A Chinese version of the IGDS9-SF was used in the current study. The internal consistency of the IGDS9-SF in the present study was excellent (α = 0.93).

#### Pittsburgh Sleep Quality Index

The Pittsburgh Sleep Quality Index (PSQI) contains 19 items rated with different forms (e.g. a Likert scale, a direct input of number) to examine an individual's sleep quality. The 19 PSQI items can be categorised into seven component scores to present the sleep quality. A higher PSQI score indicates poorer levels of sleep quality, with a cut-off >5 indicating the presence of sleep problems.^51^ A sample item of the PSQI is ‘During the past month, how would you rate your sleep quality overall?’. The PSQI has been found to have satisfactory psychometric properties in different language versions (e.g. Chinese, English, Malay, Korean).^51–55^ A Chinese version of the PSQI was used in the current study. The internal consistency of the PSQI in the present study was good (α = 0.77).

#### World Health Organization Quality Of Life Questionnaire Brief Version

The World Health Organization Quality Of Life Questionnaire Brief Version (WHOQOL-BREF) examines an individual's QoL. It has 26 items rated on a five-point Likert scale. The WHOQOL-BREF contains four domains of QoL, including physical, psychological, social and environmental QoL. A higher WHOQOL-BREF score indicates greater levels of QoL. Sample items of the WHOQOL-BREF are ‘Do you have enough energy for everyday life? (physical QoL domain)’, ‘Are you able to accept your bodily appearance? (psychological QoL domain)’, ‘How satisfied are you with the support you get from your friends? (social QoL domain)’ and ‘How satisfied are you with the conditions of your living place? (environment QoL domain)’. The WHOQOL-BREF has been found to have satisfactory psychometric properties in different language versions (e.g. Chinese, English, Portuguese, Persian).^56–61^ A Chinese version of the WHOQOL-BREF was used in the current study. The internal consistency of the WHOQOL-BREF in the present study was overall good (α = 0.79 for physical QoL; 0.84 for psychological QoL; 0.83 for social QoL and 0.85 for environment QoL).

### Data analysis

All of the statistical analyses performed in the present study were conducted with SAS 9.4 for Windows (SAS Institute, Cary, North Carolina). Descriptive statistics (including mean, frequency and prevalence) were initially performed to summarise the demographics and clinical characteristics of the participants. The associations between PIU (including problematic smartphone use, problematic social media use and problematic gaming), sleep quality and different types of QoL were assessed with Model 4 in Hayes’ PROCESS macro.^62^ Moreover, mediated effects of sleep quality on the associations between PIU and QoL were examined with the same model. A total of 12 mediation models were constructed with three independent variables (i.e. problematic smartphone use, problematic social media use and problematic gaming) and four dependent variables (i.e. physical, psychological, social and environment QoL). All mediation models have controlled age, gender, hepatitis B virus, hepatitis C virus, HIV and drug use duration. The bootstrapping method with 5000 resamples was used to examine whether the mediated effect of sleep quality was supported. Specifically, the mediated effect is viewed as significant when the 95% lower limit confidence interval (LLCI) and upper limit confidence interval (ULCI) calculated using the bootstrapping method does not include 0.^63^

## Results

Table 1 presents the participants’ demographics and clinical characteristics. The present sample reflects middle-aged participants (mean 42.19; s.d. 8.86 years); the majority were men (*n* = 273; 85.58%). Over half of the participants were single (*n* = 163; 51.10%); most were employed (*n* = 266; 83.39%). Slightly over a third of the participants were heroin or morphine users (*n* = 112; 35.11%), nearly half of the participants were amphetamine users (*n* = 151; 47.34%) and the rest were users of alcohol or other drugs (*n* = 56; 17.55%). The mean age of the participants when they began using drugs was 25.61 (s.d. 8.31) years. The duration of the drug usage was 16.50 (s.d. 10.69) years. Table 1 also presents descriptive statistics of the participants’ PIU, sleep quality and QoL. Specifically, the prevalence of problematic smartphone, social media and gaming usage was 14.11% (95% CI 10.29 to 17.93; *n* = 45), 2.19% (95% CI 0.59 to 3.80; *n* = 7) and 0.94% (95% CI 0.00 to 2.00; *n* = 3), respectively. The prevalence of sleep problems in the enrolled population was 56.43% (95% CI 50.98 to 61.87; *n* = 180).

**Table 1:** Participant characteristics (*N* = 319)

	Mean (s.d.) or *n* (%)	Prevalence (95% CI)
Age (year)	42.19 (8.86)	
Gender (male)	273 (85.58)	
Marital status		
Married	74 (23.20)	
Single	163 (51.10)	
Other	82 (25.71)	
Education year		
<12 years	185 (57.99)	
12–16 years	112 (35.11)	
≧16 years	22 (6.90)	
Employed (yes)	266 (83.39)	
Hepatitis B virus (yes)	23 (7.21)	
Hepatitis C virus (yes)	57 (17.87)	
HIV (yes)	24 (7.52)	
Living alone (yes)	44 (13.79)	
Drug use		
Heroin use	112 (35.11)	
Amphetamine use	151 (47.34)	
Alcohol use	56 (17.55)	
Drug onset age (year)	25.61 (8.31)	
Drug use duration (year)	16.50 (10.69)	
Problematic smartphone use^a^	15.42 (5.68)	14.11 (10.29–17.93)^a^
Problematic social media use^b^	10.08 (4.10)	2.19 (0.59–3.80)^b^
Problematic gaming^c^	13.46 (5.86)	0.94 (0.00–2.00)^c^
Sleep quality^d^	7.32 (4.51)	56.43 (50.98–61.87)^d^
Physical quality of life^e^	14.39 (2.69)	
Psychological quality of life^e^	13.06 (3.14)	
Social quality of life^e^	13.73 (2.78)	
Environment quality of life^e^	13.87 (2.50)	

a. Assessed with the Smartphone Application-Based Addiction Scale with a cut-off of >21 indicating presence of problematic smartphone use.

b. Assessed with the Bergan Social Media Addiction Scale with a cut-off of >19 indicating presence of problematic social media use.

c. Assessed with the Internet Gaming Disorder Scale Short Form with a cut-off of >32 indicating presence of problematic gaming.

d. Assessed with the Pittsburgh Sleep Quality Index with a lower score indicating poor sleep quality. Cut-off of >5 indicating presence of sleep problems.

e. Assessed with the World Health Organization Quality of Life Questionnaire Brief Version.

Table 2 shows that problematic smartphone use was significantly associated with poor sleep quality (unstandardised coefficient [B] = 0.12, 95% CI 0.02 to 0.21); subsequently, poorer sleep quality was significantly associated with lower QoL across all four domains. The mediated effects of sleep quality on the association of problematic smartphone use and QoL were all significant (B = −0.04, 95% CI −0.07 to −0.01 for physical QoL; B = −0.04, 95% CI −0.07 to −0.01 for psychological QoL; B = −0.03, 95% CI −0.05 to −0.01 for social QoL; B = −0.03, 95% CI −0.05 to −0.01 for environment QoL). Moreover, the direct effects of problematic smartphone use on QoL were all significant (B = −0.10, 95% CI −0.14 to −0.05 for physical QoL; B = −0.16, 95% CI −0.21 to −0.10 for psychological QoL; B = −0.13, 95% CI −0.19 to −0.08 for social QoL; B = −0.12, 95% CI −0.17 to −0.07 for environment QoL).

**Table 2:** Mediational effects of sleep quality on associations between problematic smartphone use and quality of life

Model path	Unstandardised coefficient (s.e.)	Standardised coefficient	LLCI, ULCI	*P*-value
Model: physical QoL (R^2^ = 0.10 [poor sleep quality], 0.45 [physical QoL])				
PSPU → poor sleep quality	0.12 (0.05)	0.15	0.02, 0.21	0.02
Poor sleep quality → physical QoL	−0.33 (0.03)	−0.56	−0.39, −0.28	<0.001
PSPU → physical QoL	−0.10 (0.02)	−0.20	−0.14, −0.05	<0.001
PSPU → poor sleep quality → physical QoL^a^	−0.04 (0.02)	−0.08	−0.07, −0.01	−
Model: psychological QoL (R^2^ = 0.10 [poor sleep quality], 0.34 [psychological QoL])				
PSPU → poor sleep quality	0.12 (0.05)	0.15	0.02, 0.21	0.02
Poor sleep quality → psychological QoL	−0.31 (0.04)	−0.44	−0.38, −0.24	<0.001
PSPU → psychological QoL	−0.16 (0.03)	−0.28	−0.21, −0.10	<0.001
PSPU → poor sleep quality → psychological QoL^a^	−0.04 (0.01)	−0.06	−0.07, −0.01	−
Model: social QoL (R^2^ = 0.10 [poor sleep quality], 0.29 [social QoL])				
PSPU → poor sleep quality	0.12 (0.05)	0.15	0.02, 0.21	0.02
Poor sleep quality → social QoL	−0.24 (0.03)	−0.38	−0.30, −0.17	<0.001
PSPU → social QoL	−0.13 (0.03)	−0.27	−0.19, −0.08	<0.001
PSPU → poor sleep quality → social QoL^a^	−0.03 (0.01)	−0.06	−0.05, −0.01	−
Model: environment QoL (R^2^ = 0.10 [poor sleep quality], 0.28 [environment QoL])				
PSPU → poor sleep quality	0.12 (0.05)	0.15	0.02, 0.21	0.02
Poor sleep quality → environment QoL	−0.23 (0.03)	−0.41	−0.29, −0.17	<0.001
PSPU → environment QoL	−0.12 (0.02)	−0.26	−0.17, −0.07	<0.001
PSPU → poor sleep quality → environment QoL^a^	−0.03 (0.01)	−0.06	−0.05, −0.01	−

All models controlled for age, gender, hepatitis B virus, hepatitis C virus, HIV and drug use duration. LLCI, lower limit of confidence interval at 95%; ULCI, upper limit of confidence interval at 95%; QoL, quality of life; PSPU, problematic smartphone use.

a. Mediation tested with the bootstrapping method with 5000 bootstrapping resamples.

Table 3 shows that problematic social media use was marginally significantly associated with poor sleep quality (B = 0.13, 95% CI −0.003 to 0.26). Poorer sleep quality was significantly associated with QoL across all four domains. Mediated effects of sleep quality on the association of problematic social media use and QoL were all nonsignificant. Moreover, the direct effects of problematic social media use on QoL were all significant (B = −0.14, 95% CI −0.22 to −0.06 for psychological QoL; B = −0.12, 95% CI −0.19 to −0.05 for social QoL; B = −0.09, 95% CI −0.16 to −0.02 for environment QoL), except for physical QoL (B = −0.05, 95% CI −0.11 to 0.01).

**Table 3:** Mediational effects of sleep quality on associations between problematic social media use and quality of life

Model path	Unstandardised coefficient (s.e.)	Standardised coefficient	LLCI, ULCI	*P-*value
Model: physical QoL (R^2^ = 0.09 [poor sleep quality], 0.42 [physical QoL])				
PSMU → poor sleep quality	0.13 (0.07)	0.12	−0.003, 0.26	0.055
Poor sleep quality → physical QoL	−0.35 (0.03)	−0.59	−0.40, −0.29	<0.001
PSMU → physical QoL	−0.05 (0.03)	−0.08	−0.11, 0.01	0.10
PSMU → poor sleep quality → physical QoL^a^	−0.04 (0.02)	−0.07	−0.09, 0.004	−
Model: psychological QoL (R^2^ = 0.09 [poor sleep quality], 0.30 [psychological QoL])				
PSMU → poor sleep quality	0.13 (0.07)	0.12	−0.003, 0.26	0.055
Poor sleep quality → psychological QoL	−0.32 (0.04)	−0.46	−0.40, −0.25	<0.001
PSMU → psychological QoL	−0.14 (0.04)	−0.18	−0.22, −0.06	<0.001
PSMU → poor sleep quality → psychological QoL^a^	−0.04 (0.02)	−0.05	−0.08, 0.004	−
Model: social QoL (R^2^ = 0.09 [poor sleep quality], 0.25 [social QoL])				
PSMU → poor sleep quality	0.13 (0.07)	0.12	−0.003, 0.26	0.055
Poor sleep quality → social QoL	−0.25 (0.03)	−0.40	−0.31, −0.18	<0.001
PSMU → social QoL	−0.12 (0.04)	−0.18	−0.19, −0.05	0.002
PSMU → poor sleep quality → social QoL^a^	−0.03 (0.02)	−0.05	−0.07, 0.003	–
Model: environment QoL (R^2^ = 0.09 [poor sleep quality], 0.23 [environment QoL])				
PSMU → poor sleep quality	0.13 (0.07)	0.12	−0.003, 0.26	0.055
Poor sleep quality → environment QoL	−0.24 (0.03)	−0.43	−0.30, −0.18	<0.001
PSMU → environment QoL	−0.09 (0.03)	−0.14	−0.16, −0.02	0.01
PSMU → poor sleep quality → environment QoL^a^	−0.03 (0.02)	−0.05	−0.06, 0.003	−

All models controlled for age, gender, hepatitis B virus, hepatitis C virus, HIV and drug use duration. LLCI, lower limit of confidence interval at 95%; ULCI, upper limit of confidence interval at 95%; QoL, quality of life; PSMU, problematic social media use.

a. Mediation tested with the bootstrapping method with 5000 bootstrapping resamples.

Table 4 shows that problematic gaming was significantly associated with poor sleep quality (B = 0.11, 95% CI 0.02 to 0.21); subsequently, poorer sleep quality was significantly associated with QoL across all four domains. Mediated effects of sleep quality on the association of problematic gaming and QoL were all significant (B = −0.04, 95% CI −0.07 to −0.01 for physical QoL; B = −0.04, 95% CI −0.07 to −0.005 for psychological QoL; B = −0.03, 95% CI −0.05 to −0.004 for social QoL; B = −0.03, 95% CI −0.05 to −0.004 for environment QoL). Moreover, the direct effects of problematic gaming on QoL were all significant (B = −0.06, 95% CI −0.11 to −0.02 for physical QoL; B = −0.14, 95% CI −0.20 to −0.08 for psychological QoL; B = −0.10, 95% CI −0.15 to −0.05 for social QoL; B = −0.09, 95% CI −0.14 to −0.04 for environment QoL).

**Table 4:** Mediational effects of sleep quality on associations between problematic gaming and quality of life

Model path	Unstandardised coefficient (s.e.)	Standardised coefficient	LLCI, ULCI	*P*-value
Model: physical QoL (R^2^ = 0.09 [poor sleep quality], 0.43 [physical QoL])				
Problematic gaming → poor sleep quality	0.11 (0.05)	0.14	0.02, 0.21	0.02
Poor sleep quality → physical QoL	−0.34 (0.03)	−0.58	−0.40, −0.28	<0.001
Problematic gaming → physical QoL	−0.06 (0.02)	−0.13	−0.11, −0.02	0.008
Problematic gaming → poor sleep quality → physical QoL^a^	−0.04 (0.02)	−0.08	−0.07, −0.01	–
Model: psychological QoL (R^2^ = 0.09 [poor sleep quality], 0.33 [psychological QoL])				
Problematic gaming → poor sleep quality	0.11 (0.05)	0.14	0.02, 0.21	0.02
Poor sleep quality → psychological QoL	−0.31(0.04)	−0.45	−0.39, −0.24	<0.001
Problematic gaming → psychological QoL	−0.14(0.03)	−0.25	−0.20, −0.08	<0.001
Problematic gaming → poor sleep quality → psychological QoL^a^	−0.04(0.02)	−0.06	−0.07, −0.005	−
Model: social QoL (R^2^ = 0.09 [poor sleep quality], 0.26 [social QoL])				
Problematic gaming → poor sleep quality	0.11 (0.05)	0.14	0.02, 0.21	0.02
Poor sleep quality → social QoL	−0.24 (0.03)	−0.40	−0.31, −0.18	<0.001
Problematic gaming → social QoL	−0.10 (0.03)	−0.20	−0.15, −0.05	<0.001
Problematic gaming → poor sleep quality → social QoL^a^	−0.03 (0.01)	−0.06	−0.05, −0.004	−
Model: environment QoL (R^2^ = 0.09 [poor sleep quality], 0.25 [environment QoL])				
Problematic gaming → poor sleep quality	0.11 (0.05)	0.14	0.02, 0.21	0.02
Poor sleep quality → environment QoL	−0.23 (0.03)	−0.42	−0.29, −0.17	<0.001
Problematic gaming → environment QoL	−0.09 (0.02)	−0.20	−0.14, −0.04	<0.001
Problematic gaming → poor sleep quality → environment QoL^a^	−0.03 (0.01)	−0.06	−0.05, −0.004	−

All models controlled for age, gender, hepatitis B virus, hepatitis C virus, HIV and drug use duration. LLCI, lower limit of confidence interval at 95%; ULCI, upper limit of confidence interval at 95%; QoL, quality of life.

a. Mediation tested with a bootstrapping method with 5000 bootstrapping resamples.

## Discussion

The present study sought to investigate how different types of PIU, including the problematic use of a smartphone, social media and gaming, were associated with QoL in people with SUD. We found that these three types of PIU were directly associated with low QoL. In the mediation analysis, sleep quality was a significant mediator between two types of PIU (i.e. excessive internet gaming and smartphone usage) and QoL. Moreover, the sample size used in the present study was sufficient for the mediation analysis, given that our statistical power was all nearly 1 for problematic smartphone use and QoL via sleep quality; between 0.77 and 0.99 for problematic social media use and QoL via sleep quality; and between 0.97 and nearly 1 for problematic gaming and QoL via sleep quality.^64^ These findings aligned with previous studies. Additionally, the main innovation of the current study was to investigate associations in people with co-occurrence of PIU and SUD, and assess the indirect pathways from such conditions through sleep quality toward QoL. Specifically, examinations of the different subscales of QoL provided detailed information on how the PIU may affect various dimensions of QoL in people with SUDs.

There was limited evidence for similar mediators in the association between PIU and QoL. Fazeli et al attempted to identify the mediating roles of stress, anxiety and depression in the relationship between internet gaming disorder, QoL and insomnia among over 1500 Iranian adolescents during the COVID-19 pandemic.^30^ Their findings were consistent with ours, showing a significant direct association between PIU and QoL. Moreover, they found that PIU was indirectly associated with QoL and insomnia, mediated by participants’ psychological status. The negative association between sleep disturbance and QoL, emphasised in our study, indicated that PIU might affect QoL through various pathways.

Gao et al examined the association between neuroticism and QoL.^65^ They found mediating effects for problematic smartphone usage and depression. They recruited 722 university students in China and found both direct and indirect associations between depression, smartphone use addiction and QoL.^65^ In fact, the mediating effect of smartphone addiction in the relationship between neuroticism and QoL is similar to our findings, which confirmed that the negative effects of PIU might influence QoL. Another study conducted by Lee et al in South Korea revealed the role of social support as a mediator between socioeconomic status and QoL.^66^ It was assessed in a sample of 404 people with alcohol use disorder. This finding was consistent with our results that QoL in people with SUD may be affected through multiple and various pathways. Further investigation of such routes may provide more information on risk factors that reduce QoL in people with addictive behaviours.

Although our study was not a population-based epidemiological study to elicit the sociodemographic profile of people affected by PIU and SUDs, our findings are congruent with such types of studies showing that males, younger age and misusers of central nervous system stimulants like amphetamines may be at higher risk of PIU.^16,67^ Also, the prevalence of problematic smartphone application usage may be higher than other types of PIU, such as social media or online gaming.^13,16^ Similar to the findings of previous studies, we found that nearly half of people with addictive behaviours may also have sleep disturbances.^24,27,32^ However, we also discovered a non-significant indirect association for the mediating role of sleep quality between social media addiction and QoL. One explanation for this finding may be related to the levels of emotional reactions for each type of PIU. For example, the level of excitement for gaming and smartphone usage may be greater than social media usage;^68^ hence, they cause sleep disturbance, whereas social media usage does not.

The current study was innovative (and, to the best of our knowledge, the first) in assessing several intercorrelated variables, including QoL, PIU and sleep quality in people with SUD. Nevertheless, it had several notable limitations relevant to interpreting the findings. First, we used a convenience sample in this study; therefore, we cannot generalise the results to other people with PIU and SUD. Further investigations using different sampling methods, such as snowball sampling in people with different socioeconomic and cultural statuses, are needed to better understand the potential pathways in such relationships. Second, the mediator (sleep quality) was assessed with a self-report questionnaire. Future studies should consider using a more objective assessment to validate sleep quality. Third, we tried to include a wide range of people with different types of PIU in our sample. However, the current study did not include other types of PIU, such as cybersex addiction, net compulsions and online relationship addiction. We recommend that other types of PIU should be investigated in future studies. Fourth, the majority of participants in the current study were male. As a result, the findings may not be applied to the female population. Fifth, we only excluded people with serious psychological problems, such as dementia, schizophrenia and substance-induced psychotic disorders. However, other mental health issues, such as major depression or severe insomnia, may also confound our findings and should be considered as exclusion criteria in future studies. However, we rechecked our medical records for this study and did not find any participants with these problems. Thus, we believe these other health issues would not have significantly affected our findings. Finally, as a cross-sectional study, the causality should be interpreted with caution.

In conclusion, our results showed that sleep quality might partly explain the association between PIU and QoL in people with SUDs. Therefore, sleep quality can be considered a mediator in enhancing QoL in this population. Likewise, programmes to improve sleep quality in people with SUDs should be developed in clinical settings for this population. For example, setting a schedule for regular sleep among this population may reduce the negative effects of PIU on QoL. Also, providing facilities that encourage good sleep in this group (i.e. a private and calm sleeping room or the ability to shower before sleep) may help to decrease sleep disturbances and promote their QoL. Furthermore, health promotion programmes addressing different types of PIU are needed to enhance sleep quality and QoL in people with SUD. Further assessments of other mediators may provide additional details about various underlying mechanisms that will help healthcare professionals find alternative solutions to support people with PIU and SUD.

## Data Availability

The data that support the findings of this study are available from the corresponding author, C.-Y.L., upon reasonable request.
